# Neuropsychiatric Effects of Antiviral Drugs

**DOI:** 10.7759/cureus.9536

**Published:** 2020-08-03

**Authors:** Nicholas Zareifopoulos, Maria Lagadinou, Anastasia Karela, Ourania Kyriakopoulou, Dimitrios Velissaris

**Affiliations:** 1 Department of Psychiatry, University of Patras, School of Health Sciences, Patras, GRC; 2 Emergency Department, General University Hospital of Patras, Patras, GRC; 3 Department of Internal Medicine, University of Patras, School of Health Sciences, Patras, GRC

**Keywords:** central nervous system, antiviral, efavirenz, peripheral neuropathy, monoamine oxidase

## Abstract

The adverse events of antiviral drugs are dose-dependent and often reversible. The nervous system is often affected and to date, many studies have been published regarding the central nervous system toxicity of antiviral agents. They may cause significant neuropsychiatric complications, which range from mild symptoms such as irritability and difficulty sleeping to severe complications such as depression, psychosis, and painful peripheral neuropathy, side effects which may necessitate discontinuation of treatment. The pathogenetic mechanisms may involve molecular targets common to other centrally active drugs, including human monoamine oxidase‐A (MAO‐A), serotonin receptors, gamma-aminobutyric acid (GABA) GABA-A receptors, 5-HT2A and 5-HT2C receptors and others. Notable examples include oseltamivir which may act as MAO inhibitor and efavirenz, which has an affinity for serotonin 5-HT2 and GABA-A receptors, the serotonin transporter, the MAO enzyme, and the vesicular monoamine transporter, with subjective effects which may be similar to those of the psychedelic hallucinogen lysergic acid diethylamide (LSD). Other antiviral drugs with prominent nervous system effects include nucleoside reverse transcriptase inhibitors, which are associated with the development of peripheral neuropathy after prolonged use (an effect strongly associated with older drugs which have since fallen into disfavor such as stavudine) and interferons, which may cause depression. Clinicians should be familiar with such adverse effects in order to recognise them promptly once they occur and manage them appropriately.

## Introduction and background

The adverse effects of antiviral drugs are dose-dependent and often reversible. The most common side effects include flu-like symptoms and hematologic abnormalities such as anemia and neutropenia. Moreover, some antiviral drugs cause significant neuropsychiatric complications, which range from irritability to severe depressive syndrome, including depression, cognitive impairment, and sleep disturbance.

Generally, neuropsychiatric side effects are defined as new neurological or psychiatric symptoms that develop during treatment or worsening of preexisting neurological or psychiatric disorders. It may be difﬁcult to determine whether the clinical phenotype can be attributed to the viral illness itself, the immune response to it, or the drugs used to treat it.

This paper reviews the neuropsychiatric effects of all available antiviral medications. We focus mostly on the mental health and central nervous system (CNS) effects of these medications but also discuss, in some cases, peripheral neurotoxicities such as neuropathy and myopathy. By being aware of the neuropsychiatric side effects of antivirals, clinicians are able to tailor treatment more effectively to individual patients.

## Review

Methods of literature search

The initial literature search was conducted via the PubMed MEDLINE and Google Scholar databases. Data for each drug-class, as well as for notable individual agents, were searched separately using multiple search strings (e.g., ribavirin AND neurotoxicity, efavirenz psychiatric effects, efavirenz AND psychosis, acyclovir AND nervous system). The search retrieved a total of 7,289 citations, which were narrowed down to 236 after initial screening. The final review was based on 123 articles, of which 50 (the ones most likely to be of interest to practicing clinicians regardless of specialty) were referenced in the text. We included case reports, case series, and reviews regarding adverse neuropsychiatric antiviral agent effects in the report, as well as randomized trials and preclinical ﬁndings related to possible therapeutic applications of antivirals, with the intent of providing a balanced overview of the literature on neuropsychiatric effects of antiviral drug that may be relevant to clinicians regardless of specialty. Unavailable articles, articles not in English, and book chapters that were not peer-reviewed were excluded, as were preclinical studies focusing on possible mechanisms of action for neuropsychiatric adverse effects with minimal clinical relevance. Reports of the clinical trial or observational study findings that did not present data on the nervous system adverse effects of the drugs were also excluded. The initial screening was performed independently by all authors. 

Neuropsychiatric effects of antiviral therapy in influenza

Oseltamivir and zanamivir are neuraminidase inhibitors, used for treating influenza by shortening the duration of the disease, relieving the symptoms and reducing the complications and transmission of influenza [1]. Neuraminidase inhibition prevents the virus from spreading within the respiratory tract and infecting new cells.

Results from studies in the USA, Spain, Japan, China and South Korea associate oseltamivir with many neuropsychiatric adverse events and abnormal behaviors such as delusions and perceptual disorders, delirium and delirium-like events, frightening episodes, abrupt anger, delirious speech, jumping or falling from a height, depressive episodes, mania, suicidal feelings [1, 2]. Oseltamivir phosphate (OP) is an ethyl ester pro-drug requiring ester hydrolysis for conversion to the active form of the neuraminidase inhibitor oseltamivir carboxylate (OC). Oseltamivir inhibits human monoamine oxidase‐A (MAO‐A), which is related to excitatory behaviors. Regarding acute and chronic psychotic reactions, receptors such as GABA-A, GABA-B, N-methyl D-aspartate (NMDA), and Na+, and Ca2+ channels are thought to be other candidates for investigation. Another study reported that oseltamivir sialylates a serum glycolipid that stimulates D2 dopaminergic receptor. This mechanism is related to abnormal behavior reported in some children taking oseltamivir. Unchanged oseltamivir phosphate is suggested to be related to central nervous system reactions such as acute behavioral change, occurring in the early phase of treatment [3].

In March 2007, the Japanese Ministry of Health, Labour and Welfare (MHLW) warned against the use of oseltamivir in children aged 10-19 years because of the possible cause of abnormal behavior. The U.S. Food and Drug Administration (FDA) added a warning to the oseltamivir label in 2006 to draw attention to the risk of neuropsychiatric adverse events (NPAEs). However, a causal association between oseltamivir use and abnormal behaviors or sudden death has not been established and a number of publications suggested that there is no evidence, nor plausible mechanism of action, to link oseltamivir with neuropsychiatric adverse events. They concluded that the risk of abnormal behavior was increased by influenza, and not by oseltamivir use [4]. As far as the use of zanamivir is concerned, no major neuropsychiatric adverse events have been reported.

Neuropsychiatric effects of antiviral therapy in herpes and cytomegalovirus (CMV)

Aciclovir, valacyclovir, famciclovir are medications used for the treatment of herpes infections. Since the introduction of acyclovir, in the early 1980s, neuropsychiatric adverse events have been related to the use of it. The most common neuropsychiatric adverse events, which are reported are tremor, visual and auditory hallucinations, confusion, and coma. Most cases have been associated with acute and chronic renal failure, severe illness, and the simultaneous use of other neurotoxic drugs [5]. However, there are cases with neuropsychiatric adverse events, related to acyclovir with normal renal function [6]. The neurotoxicity of acyclovir is usually described within the first 24 to 72 hours of treatment and there is complete recovery within two to seven days after treatment cessation.

Valacyclovir is an L-valyl ester and oral pro-drug of acyclovir converted to acyclovir and L-valine by first-pass intestinal and/or hepatic metabolism. Αt the highest rate (89%) acyclovir is excreted in the urine. Valacyclovir is more efficiently absorbed and increases serum concentrations more rapidly than acyclovir and is effective with less frequent administration. The neuropsychiatric adverse events related to valacyclovir include confusion, hallucination, disturbances of consciousness, ataxia, dysarthria, death elusions, psychosis, and mania. Valacyclovir toxicity occurs within 72 hours of treatment and recovery after four days after the treatment is discontinued [7].

In many cases, with neuropsychiatric adverse events related to the use of both acyclovir and valacyclovir, high serum and cerebrospinal fluid (CSF) concentrations of 9-carboxymethoxymethylguanine (CMMG) have been described [7]. CMMG is an acyclovir metabolite. In renal failure, a great proportion of acyclovir is metabolized to 9-carboxymethoxymethylguanine (CMMG), probably through the action of alcohol dehydrogenase (ADH) and aldehyde-dehydrogenase (ALDH) [5].

Acute psychosis has been reported in an AIDS patient with mild renal dysfunction who was administered intravenous gancyclovir. The psychosis occurred after 15 days of administration. Cessation of the gancyclovir and administration of haloperidol resulted in recovery. Most patients with psychosis due to valacyclovir’s structural analogs, acyclovir and gancyclovir, occur in elderly, immune-compromised patients with poor renal functioning, who receive the medication by the intravenous route [8].

Regarding famcyclovir use, no major neuropsychiatric adverse events are described. Headache (9-23%), migraine (<3%), and paresthesia (<3%) are occasionally reported. Foscarnet (trisodium phosphonoformate), an investigational pyrophosphate analog is increasingly used to treat refractory cytomegalovirus retinitis and mucocutaneous herpes simplex virus infections in immunocompromised patients. Foscarnet has been reported to cause abnormalities in serum calcium and phosphate, including cases of fatal hypocalcemia. Neurological adverse events, related to the use of foscarnet are mainly attributed to hypocalcemia [9].

Neuropsychiatric effects of antiviral therapy for chronic hepatitis B (CHB)

Chronic hepatitis B requires long-term treatment with antiviral drugs, so side effect burden and its effects on treatment adherence may be as important as the efficacy of the drugs used. Antiviral agents indicated for the treatment of hepatitis B include lamivudine, telbivudine, and entecavir (classified as nucleoside analogues), and the nucleotide analogues adefovir dipivoxil, tenofovir disoproxil fumarate and tenofovir alafenamide [10].

Lamivudine and telbivudine have been associated with myopathy and peripheral neuropathy, with the incidence of these effects being similar across all age groups and both genders [10]. The proposed mechanism underlying this reaction is the depletion of mitochondrial DNA [11]. An interesting report of two cases of lamivudine induced dystonia has been reported, which responded to prompt administration of anticholinergic agents and did not recur after lamivudine discontinuation [12]. The clinical presentation was similar to the acute dystonic reactions associated with antipsychotic drugs, which are mediated by dopamine D2 receptor antagonism in the striatum. At present, there is no evidence that lamivudine interacts with dopamine receptors, so this could be regarded as an extremely rare idiosyncratic reaction.

In another study, three of six patients with lamivudine or telbivudine­associated myopathy had a complaint of numbness in the distal end of limbs, suggesting peripheral neuropathy. The presence of neuropathy was confirmed by the electrophysiological studies and nerve biopsies by the study team. Out of 3,500 patients who received telbivudine monotherapy in clinical trials, 10 (0.28%) were reported to have peripheral neuropathy compared to nine of 48 (18.75%) patients who received combination therapy of pegylated interferon and telbivudine. Moreover, patients receiving telbivudine treatment, suffer from weakness in upper and lower extremities [10]. Myopathy is characterized by creatine kinase (CK) elevation alongside muscle pain and weakness. CK elevations are among the well­described adverse effects of nucleoside analogues (NAs), but they are not specific for myopathy and may be associated with strenuous exercise and many other illnesses. In addition, fatigue and headache were reported as the most frequent adverse events associated with telbivudine use.

Entecavir is a highly selective guanosine nucleoside analogue, approved by the FDA at a dose of 0.5 mg in treatment-naive and 1 mg/d in lamivudine-­resistant CHB patients in 2005. Psychiatric adverse events are rare in the published reports. The most frequent neuropsychiatric adverse events in clinical trials were headache (17%­-23%), fatigue (10%­-13%), and dizziness (9%), while 1-10% of patients treated with entecavir suffer from insomnia. Entecavir­-associated myopathy and peripheral neuropathy cases were very rarely reported in the literature. Patients receiving entecavir presenting with severe lactic acidosis, complain of weakness, reduced general physical condition, and impaired consciousness [10]. 

Neuropsychiatric effects of antiviral therapy in hepatitis C

The management of patients with hepatitis C is very complex. Psychiatric symptoms during antiviral therapy are reported in 30-40% of chronic hepatitis C (CHC) patients treated for 6-12 months and are much more troublesome due to their insidious onset, unpredictable nature, and potentially serious consequences.

The combination of pegylated interferon and ribavirin was the mainstay of treatment for hepatitis C infection until the approval of direct-acting antivirals. The drugs were not well tolerated and psychiatric disturbances were a common adverse event during treatment. Interferon is notorious for its depressogenic potential and patients treated with it commonly experienced apathy, anhedonia, loss of motivation, and depressed mood [13]. The use of ribavirin in combination with either interferon or direct-acting antivirals may be associated with a greater incidence of psychiatric disturbances compared to monotherapy [14].

Depression is among the most common treatment-limiting side effects of treatment with the combination of interferon and ribavirin [13]. However, clinically signiﬁcant neurological toxicity is reported in less than five percent of treated patients [15].

The recent approval of highly effective and well-tolerated direct-acting antiviral (DAA) regimens has dramatically changed the approach to hepatitis C virus (HCV) infection management. New antivirals have been developed that directly inhibit HCV, are associated with high sustained virologic response (SVR) rates and better patient tolerance [14].

There is a relative paucity of data on the adverse effects of DAAs due to their relatively recent approval. An initial increase in the Beck depression inventory score was reported in one study upon the initiation of DAA treatment, but the scores normalized by the end of the study period (12 weeks) [16]. The central nervous system effects of DAAs may be more pronounced in patients with liver cancer, as one study found an association between their use and electroencephalogram abnormalities [17]. 

Currently available data show that new antivirals do not have speciﬁc neuropsychiatric side effects. For telaprevir, the most common ‘‘psychiatric’’ adverse events are fatigue and insomnia. However, depression was only evaluated in one trial, with an incidence of 20-22% in all groups [18]. Regarding boceprevir, no speciﬁc additional psychiatric side effects could be observed [19]. Although two patients from different groups committed suicide, no speciﬁc information about depression has been given. Other adverse effects (AEs) included nausea, headache, and fatigue [20]. Ledipasvir was found to be associated with the occurrence of headache, insomnia, and asthenia [16].

Volpato et al. reported that some degree of neuropsychiatric impairment was observed in relation to treatment with sofosbuvir-based regimens in patients with cirrhosis, but not in post-liver transplanted patients, suggesting that the former may be more sensitive to mild sofosbuvir-based regimen neurotoxicity [17]. Specifically, trials of simeprevir/sofosbuvir demonstrate lower rates of insomnia and fatigue as compared with rates from boceprevir and telaprevir and suggested that these new agents may be better tolerated [16, 21]. Side effects such as weakness (30%-59%), headache (20%-30%), irritability (10%-16%), and depression (1%) have been reported in relation to treatment with sofosbuvir and may suggest mild neurotoxicity [21]. In patients receiving daclatasvir/sofosbuvir with ribavirin, the most common treatment associ­­ated with neuropsychiatric side effects included irritability, insomnia, asthenia, and fatigue [22]. At the end of a course of treatment with a sofosbuvir (SOF)-based regimen, patients with cirrhosis exhibited an increase in extra-slow EEG activity and a slowing in their reaction times [17].

There is relatively little data regarding the association between anxiety and treatment with DAAs. One study reported an increased incidence of anxiety with combination therapy as compared with monotherapy [23]. Preliminary data from clinical studies as well as clinical experience suggest that neuropsychiatric adverse events are relatively rare and usually transient as a result of DAA treatment for hepatitis C, but further research is required in larger patient cohorts to provide definitive information. 

Neuropsychiatric effects of antiviral drugs in HIV

Nucleoside Reverse Transcriptase Inhibitors (NRTIs)

Nucleoside reverse transcriptase inhibitors (NRTIs) have been associated with a number of adverse effects attributable to their interaction with human enzymes similar to their retroviral target [24]. These molecules are nucleoside analogues which lack a 3-OH group, which is necessary for phosphodiester bond formation and chain elongation. They are processed by the retroviral enzymes as regular nucleotides, but their incorporation into the newly synthesized DNA strand causes premature termination of transcription, preventing antiretroviral replication. In a similar manner, they affect human mitochondrial DNA polymerase γ and telomerase, inhibiting replication of mitochondrial DNA and telomere elongation, respectively [25, 26]. Inhibition of mitochondrial DNA polymerase may underlie the association of these drugs with a chronic toxidrome reminiscent of inherited mitochondrial disorders, which include peripheral neuropathy, myopathy [27]. All drugs of this category have been associated with this side effect profile, though the strength of the association varies. Didanosine and stavudine have been more strongly associated with peripheral neuropathy, whereas zidovudine has more commonly been associated with myopathy and myelotoxicity. The pro-drug tenofovir alafenamide, which leads to greater concentrations of the active compound in lymphoid tissue compared to other cells, may be better tolerated than the initial formulation of tenofovir disiproxil fumarate as well as most other NRTIs, though it is not devoid of such side effects.

Zidovudine is the first drug approved for the treatment of HIV and was a vital component of a first-line treatment since the 1990s. It has been associated with peripheral neuropathy attributed to depletion of mitochondrial DNA by the mechanism that is common for all NRTIs. The risk of neuropathy seems to be dependent upon the total cumulative exposure to the drug, with individuals undergoing a prolonged course of treatment at high doses being at greater risk. The effect seems to be reversible, with gradual symptom improvement following zidovudine discontinuation [28]. In contemporary clinical settings, the incidence of peripheral neuropathy or myopathy during treatment with zidovudine could be managed by drug discontinuation, given the availability of alternative treatment options [29].

Tenofovir disiproxil fumarate is the first-line treatment for both HIV and hepatitis B virus (HBV) infection, due to its similar efficacy and significantly improved safety profile compared to other NRTIs. Peripheral neuropathy is less common with tenofovir compared to other NRTIs (especially stavudine), although it does occur. Eight percent of participants developed sensory neuropathy in a South African cohort of 120 treatment-naive individuals [30]. Psychiatric adverse effects appear to be infrequent with the use of tenofovir, although the risk may be greater when it is used in combination with efavirenz. A report of nine cases of neuropsychiatric complications arising following tenofovir initiation in individuals on efavirenz containing regimen suggests the need for further monitoring and extra caution when these two drugs are to be combined [31]. The risk of these adverse effects may be reduced by the use of the more selective pro-drug tenofovir alafenamide, though further research is warranted to determine whether the difference in the toxicity profile of the two formulations is clinically significant [32].

Non-nucleoside Reverse Transcriptase Inhibitors (NNRTI)

Efavirenz is a non-nucleoside reverse transcriptase inhibitor (NNRTI), and a common component of highly active antiretroviral therapy (HAART) used to treat HIV infection. It is commonly used in combination with two nucleoside reverse transcriptase inhibitors, and it is available in a fixed-dose combination with emtricitabine and tenofovir, but tablets containing only efavirenz (to be used in combinations with other antiretroviral drugs) are also widely available. In the developed world, it is no longer considered a first-line choice for HAART due to its psychiatric side effects, which may limit patient compliance. Since the drug was approved, it has been known to produce a variety of psychiatric issues, including sleep disturbances (insomnia, nightmares), psychotic symptomatology (visual hallucinations, derealization, depersonalization), and mood dysregulation (precipitation of mania upon initiation, depression with chronic use) [33,34]. Initially, the mechanism for these effects was unknown though it was obvious that they were dose-dependent and that tolerance to the acute psychotropic effects of the drug developed a few weeks after treatment initiation [35]. Abuse of efavirenz has also been reported, primarily in South Africa where crushed tablets of efavirenz were smoked in a combination of drugs (including marijuana and heroin) called whoonga [36]. Recent evidence from in vitro and animal studies indicate that efavirenz is a potent psychotropic drug with significant affinity GABA-A receptors, 5-HT2A, and 5-HT2C receptors, while it also functions as a monoamine oxidase inhibitor and a dual serotonin/dopamine reuptake inhibitor [37]. Efavirenz may also deplete mitochondrial DNA via a mechanism similar to NRTIs, though the clinical relevance of this is overshadowed by the psychotropic side effects which occur immediately upon treatment initiation. Behavioral studies in mice indicate that it may partially substitute for LSD and methylenedioxymethamphetamine (MDMA), suggesting it has a similar effect. Neuropsychiatric adverse events, including sleep disturbances, depression, suicidality, and hallucinations have been reported in all clinical studies of efavirenz, are far more likely to occur with efavirenz compared with other antiretroviral drugs and limit regimen tolerability dramatically, being the most common reason for discontinuation.

Nevirapine is another non-nucleoside reverse transcriptase inhibitor, which, unlike efavirenz, has been infrequently associated with neuropsychiatric adverse events. A series of three case reports [38] of psychotic symptomatology and mania attributed to nevirapine have been published, but such events have been exceedingly rare and do not appear to be related to off-target interactions of the drug. Nevirapine has, however, been associated with hepatotoxicity and peripheral neuropathy, similar to nucleoside reverse transcriptase inhibitors, as evidenced by the findings of a cohort study of patients on HAART. It remains, however, unclear whether the mechanism (inhibition of mitochondrial DNA polymerase γ) is the same [39].

Protease Inhibitors

Saquinavir was the first agent of this class to be approved in 1995 with ritonavir following in 1996. Protease inhibitors may be used as a component of HAART instead of a non-nucleoside reverse transcriptase inhibitor, whereas they may also be used alone for maintenance therapy once a sustained virologic response has been achieved. As a class, they are notorious for pharmacokinetic interactions as ritonavir is a potent inhibitor of CYP3A4, potentiating the effect of drugs dependent upon CYP3A4 metabolism for inactivation. They have been associated with a lipodystrophy syndrome characterized by insulin resistance, dyslipidemia, central adiposity, and an increased risk of cerebrovascular disease [40].

Prolonged use may contribute to the development of dementia by promoting the pathogenesis of cerebral small vessel disease via this mechanism. Abuse of ritonavir has also been reported, especially in combination with other drugs of abuse, which may be potentiated by CYP3A4 inhibitors [41, 42]. Protease inhibitors have poor blood-brain barrier (BBB) penetrance, so CNS adverse effects are unlikely. They have, however, been associated with peripheral neurotoxicity, including perioral paraesthesia, taste alteration, and hearing loss. These effects may be mediated by depletion of neurotrophic factors secreted by macrophages at sensory ganglia. Ritonavir at therapeutic doses has been strongly associated with such adverse effects, but these appear to be much less frequent with other protease inhibitors [43].

Integrase Inhibitors

Raltegravir was the first drug of this class to be approved in 2007, with elvitegravir and dolutegravir being approved in 2012 and 2013, respectively. They inhibit retroviral integrase, the enzyme which enables the integration of the viral DNA transcript (synthesized via reverse transcription) into the host cell genome. It is hypothesized that they may have an improved tolerability profile compared to reverse transcriptase inhibitors due to limited off-target interactions [44]. They are among the most well-tolerated antiretroviral agents, although they have also been associated with adverse psychiatric effects, especially dolutegravir.

Side effects which have been reported include insomnia, hallucinations, abnormal dreams, mood disturbances (mostly depression), fatigue, and confusion, similar to efavirenz. Integrase inhibitor-containing regimens are, however, far less likely than efavirenz containing HAART regimens to be associated with neuropsychiatric side effects [45]. In prospective clinical trials [46] discontinuation due to such effects was low (> 1%) [47], though in cohort studies which reflect actual clinical practice, they appear to lead to treatment discontinuation more frequently and may contribute to decreased treatment adherence [48]. The association between dolutegravir and psychiatric adverse events is stronger than for the other integrase inhibitors, though the reason for this remains unknown. No studies have been published providing insight into the mechanisms of integrase inhibitor-induced neuropsychiatric events. Sleep disturbances due to integrase inhibitors may be partially attenuated by advising patients to take the drugs during the day [49].

Entry and Fusion Inhibitors

Among the most recently approved antiretrovirals are maraviroc and enfuvirtide, two agents belonging to this class. They are used in conjunction with other components of HAART. They are not first-line agents (mostly reserved for salvage therapy) and appear to be better tolerated compared to other antiretroviral drugs. They are well tolerated and have not been associated with treatment-limiting neuropsychiatric adverse effects. Substitution of efavirenz with one of these drugs may reduce the risk of neuropsychiatric effects, whereas substitution of an NRTI by a fusion inhibitor may partially attenuate peripheral neuropathy and myopathy without compromising treatment efficacy [50]. Further research is, however, needed to determine whether these drugs would be similarly well tolerated if they were used at a rate similar to first-line antiretrovirals. A summary of the data presented in the article is available in the form of a table (Table 1).

**Table 1:** Overview of neuropsychiatric effects associated with antiviral drugs MAO - monoamine oxidase; GABA- gamma-aminobutyric acid; 5-HT- 5-hydroxytryptamine; NPAE - neuropsychiatric adverse effects; BBB - blood brain barrier

Drug class	Common neuropsychiatric side effects	Mechanism of neurotoxicity	Clinical relevance	Notes
Neuraminidase inhibitors	Irritability, psychosis, mania, more commonly in children	Unclear; evidence regarding MAO inhibition or monoaminergic modulation inconclusive	Contraindicated in children < 12 years old. Use in individuals with a history of psychiatric disturbances only if benefits clearly outweigh risk.	Relevant only for oseltamivir.
Antiehrpetic drugs (acyclovir, ganciclovir and analogs)	Hallucinations, confusion, acute psychosis with acute onset	Accumulation of neurotoxic metabolites which are normally excreted in the urine (carboxymethoxymethylguanine)	Neurotoxicity more pronounced in patients with renal failure. If absolutely indicated in this population, psychiatric disturbances can be treated symptomatically.	
Foscarnet	Altered mental status, perioral paresthesia	Electrolyte disturbances, particularly hypocalcemia	Monitor and correct electrolyte abnormalities.	
Ribavirin	Depressed mood, irritability, anxiety, sleep disturbances, sexual dysfunction - gradual onset	Unclear	Consider treatment discontinuation (direct-acting antiviral agents preferred) or, if not feasible, psychiatric consultation, addition of an antidepressant.	Used in combination with interferon or direct-acting antivirals – not clear whether side effects are attributable to ribavirin alone.
Direct-acting antiviral agents for hepatitis C	Depression, insomnia, irritability, anxiety	Unclear	Address symptoms if necessary (psychotherapy, initiation of an antidepressant).	Boceprevir based regimens may be more prone to causing NPAE compared to sofosbuvir-based regimens. Concurrent ribavirin use may exacerbated NPAEs.
Nucleoside / Nucleotide analogues	Peripheral neuropathy and myopathy. Idiosyncratic manic or psychotic reactions to treatment initiation or dose adjustment	Depletion of mitochondrial DNA. More pronounced with older agents (stavudine, zidovudine) compared to the ones currently recommended (lamivudine, tenofovir)	Peripheral neuropathy is more pronounced with increased duration of exposure and higher dosage. Consider discontinuing the offending drug. For idiosyncratic reactions, discontinue offending agent, treat symptomatically.	The mechanism for idiosyncratic adverse events (psychosis – mania due to zidovudine, dystonia due to lamivudine) is unclear.
Efavirenz	Hallucinations, mood dysregulation, abnormal dreams, abuse potential, the effect may be similar to psychedelic hallucinogens – most pronounced upon initiation of treatment	5-HT2A antagonism, serotonin and dopamine reuptake inhibition, MAO inhibition, GABA-A receptor modulation	NPAEs are very common, switching to an alternative agent may lead to the resolution of symptoms. If abnormal dreams are prominent avoid dosing at night. In patients with a psychiatric history or substance abuse, alternative agents may be preferred.	Psychotropic effects likely present in all patients to some degree.
Protease inhibitors	Prolonged use may predispose to the development of dementia, cerebrovascular disease. Peripheral neuropathy	Direct psychotropic effects unlikely, may cause NPAEs via promoting lipodystrophy, diabetes, endocrine dysregulation. Depletion of neurotrophic factors is peripheral autonomic ganglia. Do not cross BBB	Avoid older drugs, use with caution in patients with multiple vascular risk factors.	Older agents (saquinavir, ritonavir) more prone to causing adverse effects compared to newer drugs.
Integrase inhibitors	Sleep and mood disturbances	Unknown	Among the most well-tolerated antiretroviral drugs. If sleep disturbances are prominent, advisable to avoid dosing at night.	Dolutegravir may be more prone to causing NPAEs than other integrase inhibitors
Entry and fusion inhibitors	No significant association with NPAEs	Unknown	May be used in place of efavirenz if it not tolerated due to NPAEs.	

## Conclusions

Neuropsychiatric effects of antiviral drugs are a common occurrence which complicates treatment. From the data presented in this article, it can be concluded that antivirals may affect the central nervous system, though for most drugs the reaction is idiosyncratic and the mechanisms are still unclear. Efavirenz has been associated with undesirable psychotropic effects similar to those observed with the use of psychedelic drugs, which are the most common reason for drug discontinuation. Further research is warranted in order to elucidate the mechanisms underlying the neuropsychiatric effects of antiviral drugs.
